# Consequences of Physical Disturbance by Tadpoles and Snails on Chironomid Larvae

**DOI:** 10.1155/2014/850782

**Published:** 2014-01-20

**Authors:** Gargi Pal, Gautam Aditya, Niladri Hazra

**Affiliations:** ^1^Entomology Research Unit, Department of Zoology, The University of Burdwan, Burdwan 713 104, India; ^2^Ecology Laboratory, Department of Zoology, The University of Burdwan, Burdwan 713 104, India

## Abstract

Indirect interactions among community members impact on organisms. The effects of two snails, banded pond snail, *Bellamya bengalensis* (Lamarck), and Red-rimmed melania, *Melanoides tuberculata* (Müller), and tadpoles of Asian common toad, *Duttaphrynus melanostictus* (Schneider), on nonbiting midge larvae, *Chironomus striatipennis* Kieffer, were observed in experimental microcosm. Decrease in tube number and tube length of midge larvae was observed compared to control condition due to introduction of selected above mentioned organisms. The direct effects of non-predator organisms on the midge larvae are due to physical disturbance that destroys their tubes. This may result in vulnerability of midge larvae to predators in the wild. So the community structure may be altered by indirect effects, where one or more species, through their direct disturbance, indirectly change the abundance of other species.

## 1. Introduction

The dynamics of in aquatic communities are influenced by direct and indirect interactions among organisms. While changes in the population of interacting species can be identified due to direct interactions like predation, herbivory, and parasitism, it is often more subtle in case of indirect interactions [1, 2]. For instance, the effects of sharing common resource may remain obscured for two competing species until the resource is limited. [3, 4]. However, instead of any numerical changes in population, the indirect effects may be realized in terms of variations in the life history features or shift in habitat utilization of the reference population within the community. Such processes have been viewed as “trait-mediated effect” and “density-mediated effect” [1, 2, 5], when the competing consumers for resource utilization influence the life history features of each other. Several empirical studies have demonstrated habitat shifting and alteration in the life history features among species as a consequence of indirect interactions [6–8]. Quantification of relative importance of direct and indirect effects in community regulation in terrestrial and aquatic habitats has been experimentally studied [9]. Following comparisons among these different communities, it is observed that direct effects are generally greater in magnitude and less variable than indirect effects, and in terms of food web, short chain indirect effects were stronger than long-chain effects. In species ensembles of aquatic communities, chironomid larvae, tadpoles, and snails exploit detritus as a resource. The feeding strategies of these organisms differ from one another. While the chironomid larvae are scrapers or gatherers, the tadpoles scrape substrates for food. The snails graze on the sediment floor. In course of forging or movement of the tadpoles and snails in the sediment, they are expected to influence the tube building and foraging behaviour of midge larvae in a density-dependent manner, through an interference competition.

The chironomids play an important role in the aquatic food web serving as a major link between producers and secondary consumers [10] and contribute to the dynamics of aquatic ecosystems on account of its numeric abundance and role in nutrient cycling. According to Lawler [11], rice fields serve as temporary wetlands, which harbour many of the same species that breed in natural ponds. Therefore, the rice agroecosystem influences the regional biodiversity of many invertebrates and vertebrates. The midge larvae are food for fish, water fowl, and other invertebrates [12–15]. The larvae of *Chironomus* spp. make tubes in the soft benthic sediments of freshwater habitats by incorporating detritus with their saliva. The tubes of chironomids are thought to have several functions. First, the tubes let larvae to obtain oxygen and food efficiently, because the larvae produce water currents in their tubes by wiggling their bodies, which act to trap food in the water at the open mouth of the tubes, and faeces can also be washed out [16]. Secondly, the tube acts as physical barrier against predaceous natural enemies [17]. Thirdly, the tubes act as protective barrier for the chironomid larvae against chemical toxicants [18]. Fourth, the tubes play an important role by contributing to a decrease in the desiccation rate [19]. 

The species assemblage of the tadpoles, snails, and midge larvae in the wetlands like rice fields, ponds, temporary pools, and marshes suggest that a possible competition could exist for exploiting the same resources in the benthic habitat. The effect of this competition would bear an impact on patch characteristics that might indirectly influence the spatial orientation and the growth and developmental pattern of chironomids. As a consequence, alteration in the foraging pattern of the predators may be anticipated affecting the search area and overall predation strategy. The present study was an attempt to highlight the possible effect of interspecific competition on the spatial orientation of the chironomid larvae. This may make it possible to justify the proposed hypothesis concerning interaction between non-predator species sharing the same trophic level as a factor for dispersion of the chironomid larvae and their possible role in susceptibility of midge larvae due to their repetitive destruction of tubes.

The objectives of the study were to observe the direct and indirect effects of two snail species, banded pond snail, *Bellamya bengalensis* (Lamarck), and Red-rimmed melania, *Melanoides tuberculata* (Müller), and tadpole of Asian common toad, *Duttaphrynus melanostictus* (Schneider), on the larvae of *Chironomus striatipennis* Kieffer in terms of tube number, tube length, and the life stages of the species. The direct effects of these species on the midge larvae are assumed to be due to competition for food and limited space, and the indirect effects are hindrance of tube construction of the chironomid larvae. As a result, midge larvae were exposed and would be vulnerable to predators. This can be viewed as an indirect effect. Indirect effects have long been a well-established observed fact in aquatic communities [20, 21].

## 2. Materials and Methods

### 2.1. Collection and Maintenance of Organisms

Egg masses of chironomid midges along with the substratum were collected from a pond within the University of Burdwan, Golapbag Campus, and the masses were put in a plastic container with little water. Separate plastic tubs (40 cm × 32 cm × 13 cm) each containing 10 g of ground soil were used as substratum. As soon as the larvae hatched, they were transferred to plastic tub. Larvae were reared after [22–24]. Intermittent aeration was made with the air pump (SOBO Aerator, SOBO, China). Around 5 mg of crushed fish food (Tokyu, Thailand) was added every alternate day to each tub. Water of the tub was replaced every 24 h. The culture was maintained in the laboratory in natural light at 32°–34°C) 7.1–7.8 pH until they attained a certain length of 2–4 mm. Some of the mature larvae (4th instar) were put individually in the glass vial with little amount of tap water (4–9 mm depth) and the substrate. These vials, plugged with dry nonabsorbent cotton, were then kept inclined on a rearing wooden stand at room temperature. Eclosion of the adults was observed daily. Newly emerged imagines were kept in a cool dark place for 24–48 hours for sclerotization of cuticle. The adults were identified as *Chironomus striatipennis* Kieffer (Diptera: Nematocera: Chironomidae: Chironominae: Chironomini).

Tadpoles of *Duttaphrynus melanostictus* (Anura: Bufonidae) (0-leg stage) were procured from the same pond and placed in plastic tubs with sufficient amount of water in the laboratory under the same conditions as above. Fresh water and crushed fish food (Tokyu, Thailand) were given every day.

The snails, *Melanoides tuberculata* (Gastropoda: Thiaridae) and *Bellamya bengalensis* (Lamarck) (Gastropoda: Viviparidae), were collected from the substratum of the pond using a dip net attached with a long handle and were maintained in the laboratory in plastic tubs containing dried sediments and dead terrestrial plant leaves.

### 2.2. Microcosm Design

Individual tub (40 cm × 32 cm × 13 cm) having 10 g of sundried and crushed soil mixed with tap water was prepared to serve as substratum for the microcosm. After hatching, approximately 300–400 chironomid larvae were added in each tub containing 5 L of water. The numbers varied depending on the egg masses. Five grams of finely ground soil was added to each tub at 10-day interval to serve as food and tube building material. As supplementary food, 20 mg of crushed fish food was added to the tubs, every day. The microcosm was left undisturbed, when the experiments were initiated. Each tub was divided into 12 equal quadrats using threads placed along the edges. End of each thread was knotted with a small stone in order to maintain the appropriate shape of the quadrat from top view (10 cm × 10.7 cm). The quadrats allowed monitoring of the tube lengths and changes in the microcosm sediment in smaller divisions. With the initial 5 L tap water being poured in the tub, 500 mL of same water was added to the tubs for consecutive nine days, so that at the time of initiation of the experiments the total volume of water was 14 L forming a microcosm with an average depth of 13 cm. Addition of water allowed maintenance of congenial condition for the growth of the midge larvae, reducing water fouling. This setup was maintained with natural light and as previously described.

### 2.3. Experimental Design

**Figure 1:**
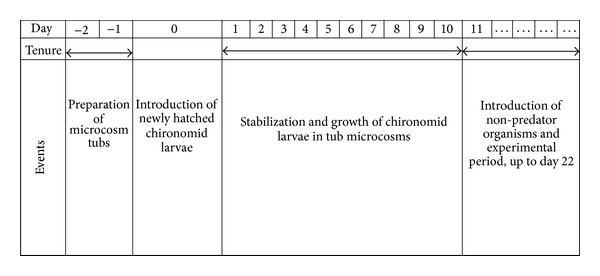
Flow chart explaining the chronology of the experimental design.

To assess the impact of the snail *T. tuberculata* and tadpoles of* D. melanostictus*—on the tube building of *C. striatipennis*, the following experiments were carried out. In all instances, the experiments were initiated on the 11th day after hatching of *C. striatipennis *followed by maintenance in the microcosms, when the larvae attained an average of 2–4 mm in length (Figure 1).

#### 2.3.1. Assessment of Effects of Snails and Tadpoles on Developing Chironomid Larvae

On the 11th day, the number of tubes in each quadrat in every tub was counted. Following this, a pair of *B. bengalensis* was added in each microcosm. Three microcosms without the snails were considered as control. On the next 24 h period, four individuals and on the third day of experiment eight individuals of *B. bengalensis* were added to the experimental replicates. In the next six consecutive days, *M. tuberculata* and tadpoles of *D. melanostictus* were added in succession following the same procedure. Thus, the experiment was continued for nine days using the three different species with a density-level variation of 2, 4, and 8.

**Figure 2:**
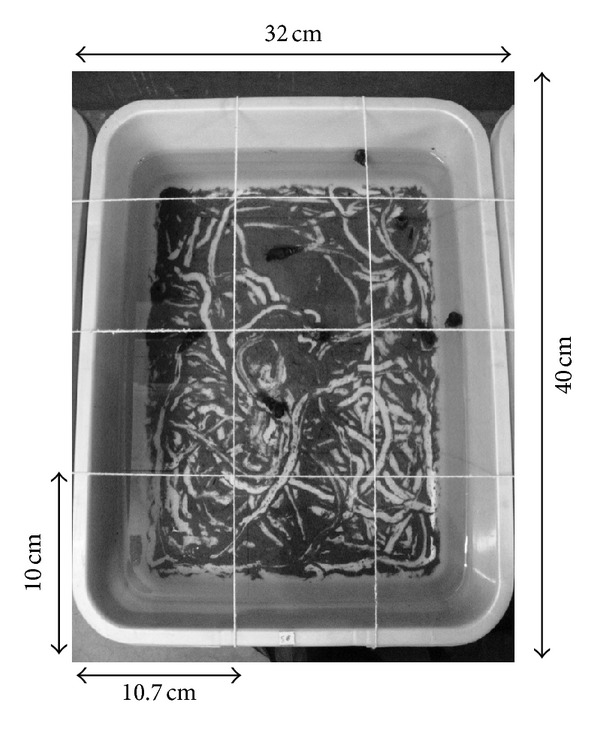
A typical “*Bunty* tub” used in the experiments, elaborating the “quadrats” from top view. The dashed lines represent the threads attached to the edges of the tub, so that from top view each quadrat (rectangular in shape—10.7 × 10 cm^2^) can be differentiated.

At the end of each 24 h period following the placement of the snails or tadpoles, the numbers of tubes in each quadrat (Figure 2) were noted using a magnifying glass. The lengths of twelve randomly selected tubes from each quadrat were measured to the nearest 0.1 mm using a transparent ruler. The data on the tube numbers and length were analyzed with a 3-way factorial ANOVA using days, quadrat, and species as variables [25, 26].

## 3. Results

The chironomid larvae constructed tubes as soon as they were introduced to the microcosms and remain distributed in the substratum. With the addition of fish food *ad libitum*, the sediments provided good nourishment for the growth of the chironomid larvae, evident from the colonization and tube formation by the 1st instar larvae.

The introduction of snails and tadpoles caused changes in the spatial distribution of the chironomid larvae. Movements of the snails and tadpoles on the substratum displaced the sediments (Figure 2) and created distinct undisturbed sediment patches with intervening trails. While in the control, these changes were absent and the distribution of the chironomid larvae was not affected as evident from the orientation of the tubes in the substratum since their construction. The trails formed by the movement of the snails were random compared to the tadpoles that exhibited a distinct pattern. In case of tadpoles, the sediments in the middle part of the microcosms were rarely affected. In contrast to this, the movement of snails affected the middle portion of the substratum more than the border areas. Continuous movement of the snails and the tadpoles led to the displacement of chironomid larvae and the changes of sizes of the tubes were apparent. In few cases, the chironomid larvae were noted to move out of the tubes or swim freely in the water. This supported the view that the movement of the snails and the tadpoles caused disturbance by destroying the tubes and consequent displacement of the chironomid larvae.

**Figure 3:**
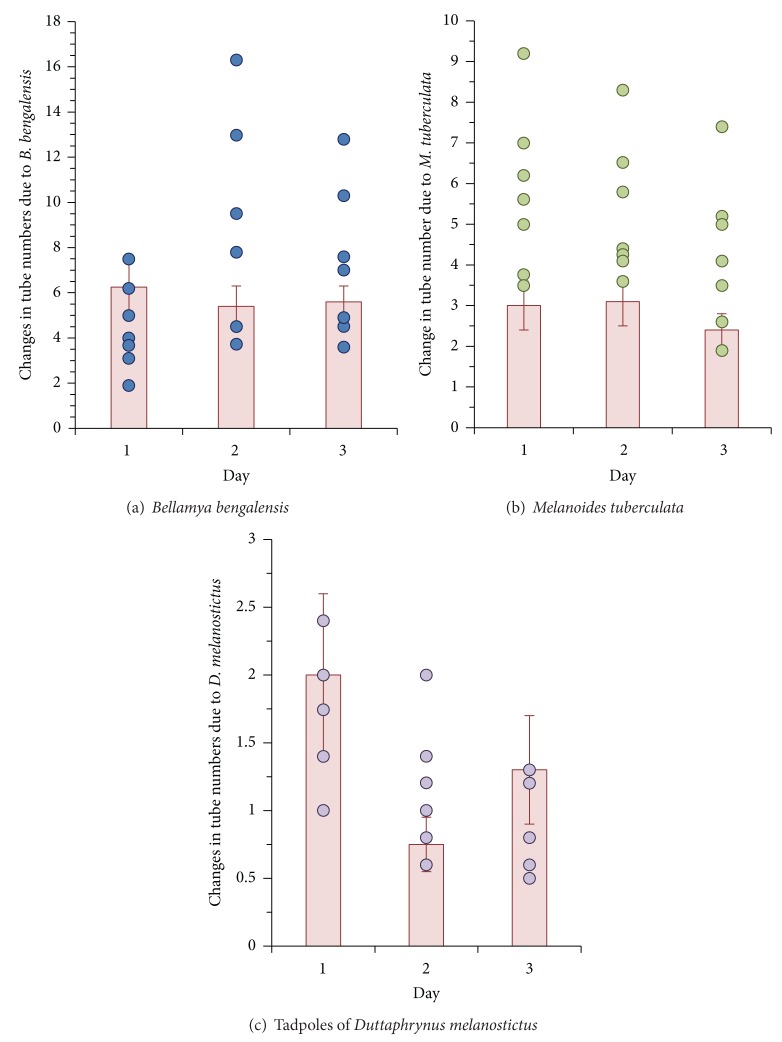
Changes in the tube numbers of the developing chironomid larvae in the quadrats due to the activity of snails and tadpoles (closed circles) and control (without non-predators, bar) microcosms.

**Figure 4:**
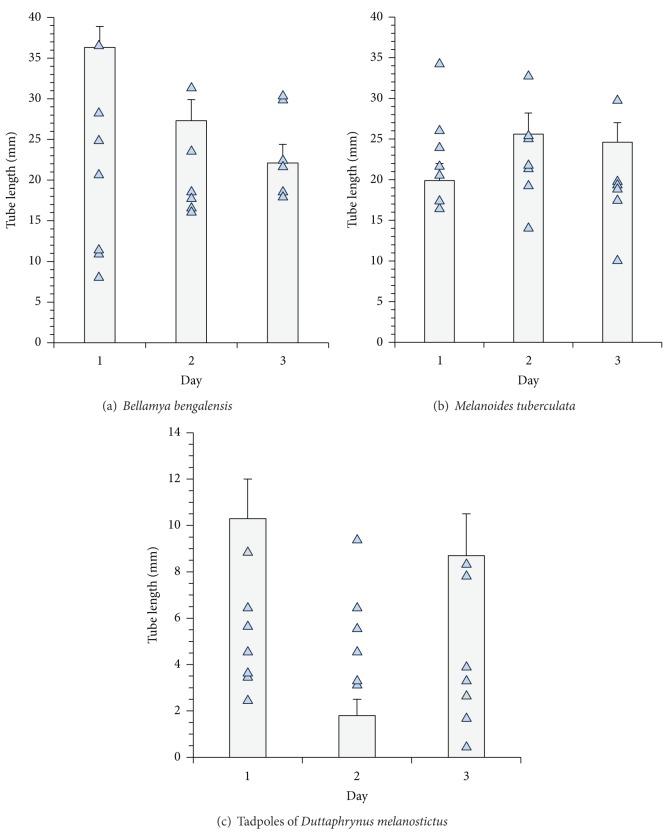
Changes in mean tube length of the developing chironomid larvae in the quadrats in the presence of snails and tadpoles (filled triangles) and control (without non-predators, bar) microcosms.

On the tenth day, the number of tubes present in each quadrat of the replicate tubs, prior to addition of the snails and tadpoles, ranged between 8 and 39 (22.4 ± 3.9 SE; *n* = 84 quadrats) and the tube lengths ranged between 6.1 and 8 mm (7.06 ± 0.23 SE; *n* = 200 tubes). In the subsequent days, following addition of snails and tadpoles in the microcosms, the changes in the tube number and tube length are shown in Figures 3 and 4. The changes in tube numbers were significantly different (Table 1) among the replicates, non-predator species, and their relative density. The differences in the replicates could be attributed to possible differences in the number of individuals present in each tub, since no counts were made following the initial inoculation with chironomid. Possible variations in the development rate of the larvae may have contributed to this difference. The post hoc Tukey test reveals significant differences between non-predator snail and tadpole species pairs as well as their relative density, indicating that the level of disturbances varies with the species and increases with density. Changes in the tube lengths of chironomid larvae in the microcosms were significantly affected (Table 2) by the disturbance by the non-predator snails and tadpoles and among the quadrats within the microcosms. Upon addition of non-predator snails and tadpoles in succession, the density effect was not significant as observed through the post hoc Tukey tests (Table 2). A positive correlation (*r* = 0.317; *P* < 0.001; df = 755) was noted in the increment of tube length with time signifying that the development of chironomid midges was not halted.

## 4. Discussion

The spatial distribution of the chironomid larvae was significantly affected due to presence of the snails and tadpoles. The density effects of these non-predators appeared to be insignificant perhaps due to the space available. Possibly owing to recurring displacements of the chironomid larvae by the non-predators, the density effects were superimposed and remained obscured as the time interval for observations was 24 h. Observations at a minor scale less than 24 h may not have allowed quantification of the density effects of these species that share the same trophic levels with the chironomid larvae. Nonetheless, it is evident that the midge larvae were displaced from the tubes by the snails and tadpoles and forced to move to the patches created due to the movements of the non-predators on the substratum. The trails along the patches formed due to the movements of the snails and tadpoles lead to the changes in the architecture of the substratum. This can be viewed as disturbance resulting in the shifting of the location of the chironomid larvae. Few chironomid larvae come out of the tubes. This observation is in conformity with the fact that the chironomids do respond to the changes in the habitat conditions either by reorienting [27, 28] or by extinction at the local scales particularly in the temporary pool [29–31] and streams [32, 33].

The snails, *B. bengalensis* and *M. tuberculata*, are obligatory detritivores and depend heavily on the grazing of the substratum or on solid detritus. The tadpoles are also reliant on the detritus, but their feeding mechanism is more of a scraper than grazer. This might be a reason why the patches formed on the substratum due to the movement of the snail species appeared different from tadpoles. While the tadpoles used more of the borders of the tubs and formed more symmetric patches, the snails produced an asymmetric pattern dividing the detritus layer into irregular patches. Moreover, the wall of the microcosm was also utilized by the snails, which was rarely found to be explored by the tadpoles. The chironomid larvae remain concealed in the tube at a fixed space with least movements [34]. In the present context, both in the experimental and the control microcosms, the patch occupancy and the movement of the midge larvae were far less compared to the those of snails and tadpoles. These observations suggest that despite the chironomids sharing the same trophic levels with the tadpoles and snails, they are restricted in using available spaces of the habitat. The tadpoles and the snails utilize a larger proportion of the habitat. In the control tubs, reorientation of chironomids was evident, possibly as a result of the density effects. Although the reorientation pattern could not be differentiated with the experimental tubs, the movement of the chironomids in the control tubs was much less. Empirical evidence suggests that, under natural conditions, chironomid larvae appear in dense number in a single patch and remain crowded [35–37]. To avoid crowding and intraspecific competitive interactions, the reorientation is a possible alternative [38]. Also predator-induced reorientation of the larval distribution of chironomids is known [39, 40]. However, in natural conditions, the environmental factors are more dynamic and can contribute to the addition of the resources at a regular interval. This was absent in the experimental conditions for the midge larvae. Nonetheless, the exploitative competitive effects from the snails and the tadpoles are manifested by the difference in the mean number of chironomids displaced from each quadrat.

The tube length in the chironomid larvae was found to be affected due to the presence of snails and tadpoles. The length of the midge larval tubes can be considered as an indicator of the disturbance caused by non-predator tadpoles and snails, since the length of the tubes reflects the size of the chironomid larvae residing there. In the present instance, the consequences of movements of the snails and the tadpoles over the tubes were evident; a direct effect on the tube length can be measured. As a consequence, the developmental pattern of the chironomids may be affected owing to this disturbance. The indirect effect of the tadpoles and snails with the chironomid larvae can have an additional effect by making the latter vulnerable to the predators and expend more time and energy for reconstructing tubes. Thus, the disturbance of snails and tadpoles can be described as interference competition that caused displacement of chironomid larvae due to destruction of tube nest. The individual larval tube length was also reduced as a consequence of the disturbance by the non-predators, thereby inducing negative effects on the chironomids.

## 5. Conclusion

The present study demonstrates consequences of indirect interactions between chironomid larvae and non-predator snails and tadpoles that share the same space resource. Repetitive movements of the snails and the tadpoles over larval habitat can be considered as a disturbance, which caused the destruction of tubes build by chironomid larvae. Rapid rebuilding of tubes is necessary to continue feeding and larval development and to avoid predation. Disturbance by non-predator snails and tadpoles decreased the length of the larval tubes. The change in numbers of tube and the decrease in tube length of chironomid larvae may be viewed as a result of indirect interactions with non-predator organism. In aquatic communities where tadpoles, snails, and chironomid coexist, such indirect interactions may influence larval development and augment vulnerability of chironomid to predators.

## Figures and Tables

**Table 1a:** (a)

Source of variation	Sum of squares	df	Mean square	*F*	*P* value
Microcosms (TRAY)	1089.6	6	181.60	9.26	0.001
Non-predator species (SP)	4198.9	2	2099.44	107.08	0.001
Density (DEN)	155.25	2	77.62	3.96	0.020
TRAY∗SP	1097.5	12	91.46	4.66	0.001
TRAY∗DEN	808.31	12	67.36	3.44	0.001
SP∗DEN	697.02	4	174.25	8.89	0.001
TRAY∗SP∗DEN	852.26	24	35.51	1.81	0.010
Error	13587	693	19.61		

Total	22486	755			

**Table 1b:** (b) Multiple comparisons between non-predator species. The Tukey HSD with standard error 0.394; df = 251,2

(*I*) SP	(*J*) SP	|*q*| Mean difference |(*I* − *J*)|	Significance
B	T	5.631	0.001
B	TH	1.7143	0.001
T	TH	−3.917	0.001

**Table 1c:** (c) Multiple comparisons between density of non-predators. The Tukey HSD with standard error 0.394471; df = 251,2

(*I*) SP	(*J*) SP	|*q*| Mean difference |(*I* − *J*)|	Significance
2	4	−1.044	0.02
2	8	−0.194	0.87
4	8	0.8492	0.08

T: tadpoles; B: *B. bengalensis*; TH: *T. tuberculata*.

**Table 2a:** (a)

Source of variation	Sum of squares	df	Mean square	*F*	*P*-value
Non-predator species (SP)	119725	2	59862.30	44.93	0.00
Density (DEN)	2288.1	2	1144.05	0.86	0.42
AREA (QUAD)	59067	11	5369.70	4.03	0.00
SP∗DEN	10044	4	2511.11	1.88	0.11
SP∗QUAD	31371	22	1425.94	1.07	0.37
DEN∗QUAD	10645	22	483.87	0.36	1.00
SP∗DEN∗QUAD	38184	44	867.82	0.65	0.96
Error	863371	648	1332.36		

Total	1134695.1	755			

SP.: species, QUAD: quadrat.

**Table 2b:** (b) Multiple comparisons between non-predator species. The Tukey HSD with standard error 3.252; df = 251,2

SP (*I*)	SP (*J*)	|*q*| Mean difference |(*I* − *J*)|	Significance
B	T	−30.44	0.001
B	TH	−19.44	0.001
T	TH	10.99	0.002

**Table 2c:** (c) Multiple Comparisons between densities of non-predator species. The Tukey HSD with standard error 3.251817, df = 252,2

SP (*I*)	SP (*J*)	|*q*| Mean difference |(*I* − *J*)|	Significance
2	4	0.00	1.00
2	8	−3.69	0.49
4	8	−3.69	0.49

T: tadpoles; B: *B. bengalensis*; TH: *T. tuberculata.*
